# Vascular Regeneration in a Basal Chordate Is Due to the Presence of Immobile, Bi-Functional Cells

**DOI:** 10.1371/journal.pone.0095460

**Published:** 2014-04-15

**Authors:** Brian P. Braden, Daryl A. Taketa, James D. Pierce, Susannah Kassmer, Daniel D. Lewis, Anthony W. De Tomaso

**Affiliations:** Department of Molecular, Cellular, Developmental Biology, University of California Santa Barbara, Santa Barbara, California, United States of America; Center for Molecular Biotechnology, Italy

## Abstract

The source of tissue turnover during homeostasis or following injury is usually due to proliferation of a small number of resident, lineage-restricted stem cells that have the ability to amplify and differentiate into mature cell types. We are studying vascular regeneration in a chordate model organism, *Botryllus schlosseri*, and have previously found that following surgical ablation of the extracorporeal vasculature, new tissue will regenerate in a VEGF-dependent process within 48 hrs. Here we use a novel vascular cell lineage tracing methodology to assess regeneration in parabiosed individuals and demonstrate that the source of regenerated vasculature is due to the proliferation of pre-existing vascular resident cells and not a mobile progenitor. We also show that these cells are bi-potential, and can reversibly adopt two fates, that of the newly forming vessels or the differentiated vascular tissue at the terminus of the vasculature, known as ampullae. In addition, we show that pre-existing vascular resident cells differentially express progenitor and differentiated cell markers including the *Botryllus* homologs of CD133, VEGFR-2, and Cadherin during the regenerative process.

## Introduction

Differentiated cells, tissues and organs must be replenished throughout adult life in response to normal growth, aging, and injury through regenerative processes. However the potential and source of regeneration vary amongst both organisms and in individual tissues [1], [2]. While it is thought that a small population of tissue-restricted, self-renewing progenitors cells carry out the process of regeneration, the cellular identity and molecular mechanisms that control sources of regeneration in many organisms and tissues are not well understood.

Here we investigate the source of the regenerative potential exhibited by the extracorporeal vasculature of the colonial ascidian *Botryllus schlosseri*. Ascidians such as *Botryllus* can be found in shallow waters and harbors throughout the world [3] and are considered to be the closest non-vertebrate relatives to the vertebrates [4]. *Botryllus* has been the focus of numerous genetic, developmental, and regenerative studies including studies of asexual development [5], [6], stem cell parasitism [7], allorecognition [8]–[11], and vascular regeneration [12]–[15]. The latter studies demonstrated that the vascular tissue of *Botryllus* is highly regenerative and will replace entire portions of differentiated tissue, on the order of several cm^2^, within 24–48 hours following surgical ablation [15].

**Figure 1:**
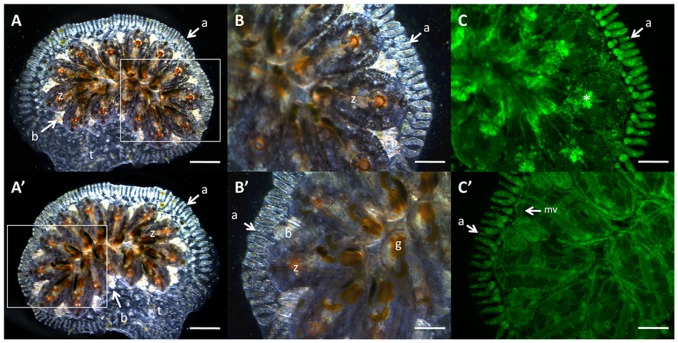
*Botryllus schlosseri* Morphology. (**A**) Dorsal and (**A′**) ventral view of adult *Botryllus schlosseri* colony showing individual adult animals known as zooids (z), asexual propagating buds (b) terminal projections of the extracorporeal vasculature known as ampullae (a) all encompassed in a cellulose based extracellular matrix known as the tunic (t). (**B**) Dorsal and (**B′**) ventral view of the inset showing detail of *Botryllus* colony including the gut (g) located in the individual zooids (z). (**C**) Dorsal and (**C′**) ventral view of GFP injected colony showing the peripheral ampullae (a) and marginal vessel (mv) which are part of the extracorporeal vasculature that connects individual zooids and buds through a common blood supply, distinguished from the auto-fluorescent pattern of pigmented cells in the zooid (asterisk). Scale bars: A = 1 mm, B–C = 500 µm.

The vascular system of *Botryllus* consists of two major structures, an internal plot of sinuses and lacunae of mesenchymal cells that surround the major organs and tissues of individual animals in the colony known as zooids, and a large extracorporeal vasculature consisting of ramified mono-layered vessels embedded within an extracellular matrix made of both cellulose and protein components, known as the tunic [13], [15]–[17] (Figure 1). The morphology of the extracorporeal vessels is inverted in comparison to vertebrate vascular structures: the vessels are not made of mesodermally-derived endothelial cells, but rather a single layer of ectodermally-derived cells which form a tube with the basal lamina lining the lumen, and the apical side of the cells facing the extracellular environment [13], [15], [16]. In addition, the extracorporeal vasculature can be subdivided into two regions. First are the vessels, which ramify throughout the colony, connecting the zooids via a common, extracorporeal blood supply. Second are terminal protrusions of these vessels, called ampullae. Ampullae are involved in multiple processes, including adherence of the colony to the substrata as well as the site of allorecognition in *Botryllus*. For the latter, when ampullae of two individuals come into contact, they initiate a reaction that results in either anastamosis of the two vasculatures at the site of contact forming a blood chimera, or an inflammatory reaction which blocks vessel fusion. Proteins involved in this reaction are differentially expressed on the tips of the ampullae and absent from the vasculature, including the fuhc^sec^ and fuhc^tm^ [9], fester [10], uncle fester [8], and HSP-40L [18]. Additionally the differentiation state of both ampullae and vessels seems to be highly dynamic and reversible as ampullae become vessels that do not express allorecognition proteins during anastomosis and reversibly vessels become ampullae that do express allorecognition proteins during regeneration [8], [10].

The regeneration of these differentiated vascular tissues has been shown to occur through a sprouting mechanism with the participation of angiogenic factors: VEGF, FGF-2, and EGF [12]. In addition, the homolog of VEGR-2 is necessary for the regeneration exhibited by vascular tissue [15]. While the regenerative potential of the extracorporeal vasculature, including vessels and ampullae tissue in *Botryllus* is clear, the cellular identity and molecular source of this regenerative potential is unknown.

The large and experimentally accessible vasculature, natural parabiosis that occurs between histocompatible colonies, and vast regeneration potential of the *Botryllus* vasculature makes *Botryllus* a unique model for studies on vascular biology. Here we use a novel vascular cell lineage tracing technique to both trace and isolate cells that participate in the vascular regeneration of the extracorporeal vasculature and differentiation of ampullae. In this study we gain insights into the bi-potentiality of these vascular tissue resident cells and determine their regenerative proliferation, mobility, as well as their cellular and molecular contributions to regenerating differentiated vascular tissue.

## Materials and Methods

### Animals

*Botryllus schlosseri* colonies were collected from the yacht harbor in Santa Barbara, CA, spawned and cultured in laboratory conditions at 18–20°C according to ([19]. Collections were performed at only one local harbor, the Santa Barbara Harbor (Longitude -119.6887448 and Latitude 34.407), which is owned by the City of Santa Barbara and performed under the authority of the California Department of Fish and Game. These collections did not involve any endangered or protected species. Colonies were developmentally staged matched based on blastogenic stage cycles according to [20]. Genetically identical stage matched sub-clones of adult colonial systems with 5–8 zooids, aged 3–6 months were used for both ampullectomy and chimaeric assays.

### Fluorophore Preparation and Injections

Recombinant GFP and mCherry fluorescent protein were produced in a BL21(DE3)pLysE (Invitrogen, C6565-03) bacterial cell line through a pET28A expression vector (Merck Millipore, 69864-3) under a lac operon expression system. The GFP and mCherry genes (Clontech) were cloned into the pET28A vector with BamHI and XhoI which were introduced through primer design of PCR with *Pfu* polymerase (Agilent Cat, 600153). The recombinant GFP and mCherry protein contained N- & C- termini 6xHis tag. Expression of recombinant GFP and mCherry was induced with a final concentration of 1 mM IPTG when bacterial culture reached an optical density at 600 nm (OD_600 nm_) absorbance of 0.5 to 0.8 at 37°C with shaking at 250 RPM. Induced cultures grew at 37°C for 18 to 24 hours with shaking at 250 RPM.

Recombinant protein was extracted from the bacteria via sonication in Equilibrium/Wash Buffer as described in the HisPur Cobalt Resin protocol (Pierce Biotech, 89964) and supplemented with 1 mM phenylmethylsulfonyl fluoride (PMSF). Bacterial debris were removed via centrifugation and extracted proteins were incubated with HisPur Cobalt Resin overnight at 4°C. Resin with bound recombinant GFP or mCherry protein was collected in a gravity flow column then washed and eluted as described in the manufacturer's protocol (Pierce Biotech, 89964). Recombinant GFP and mCherry protein was further concentrated with an Amicon column with a 3 kDa MWCO (Millipore, UFC500324). Final concentrations of protein were determined by Bradford assay following the manufacturer's guidelines (Pierce Biotech, 23200) with BSA used to generate the standard curve.

Recombinant GFP and mCherry proteins along with Fluorescein conjugated Dextran (FITC-Dextran) (3000MW) (Life Technologies, D-3305), Alexa Fluor 488 conjugated BSA (Life Technologies, A13100), and Alexa Fluor 594 conjugated BSA (Life Technologies, A13101) were diluted in PBS to a final concentration of 0.1 µg/ml. While we injected a range between 0.01 µg/ml to 1 µg/ml of these macromolecule fluorophores to label vasculature cells, our analysis indicated that 1 µl injections of 0.1 µg/ml per system (5-8 zooids) of macromolecule fluorophore per system gave the strongest vascular labeling without residual fluorophore in the blood stream at time points of 24 hours and beyond. To determine the presence of lysosomes in these tissues we used LysoTracker Green DND-26 (Life Technologies, L-7526) at 1∶100 in Seawater and soaked the animals for 30 minutes.

### Chimeric Assays and Ampullaectomy Surgeries

Chimeric assays were conducted using multiple single systems of genetically identical colonies that were injected with 1 µl of 0.1 µg/ml of either mCherry protein, FITC-Dextran, Alexa Fluor 488 conjugated BSA, or Alexa Fluor 594 conjugated BSA to label vasculature cells. Single systems were allowed to incubate 24 hours prior to additional system contact to prevent transfer of the fluorescent macromolecules and allow for cell labeling to occur. Colonies with labeled vasculatures were then placed on slides in close proximity to each other and allowed to undergo vascular anastamosis. Once common blood flow was established, chimeric animals were then allowed to undergo a takeover event prior to being separated by ampullaectomy surgery. In both single systems and chimeric colonies peripheral ampullae and parts of the marginal vessels were surgically removed by ampullaectomy surgeries as described by [15].

### In situ hybridization

Whole-mount in situ hybridization was performed with digoxigenin (DIG)-labeled probes as described by [9]. Specific antisense probes for both CD133 and VEGFR-2 were synthesized from PCR products using CD133 and VEGFR-2 clones coding for 873 bp and 348 bp regions of the respective genes. TSA-plus detection (PerkinElmer, NEL753001KT) with Cyanine 3 (CD133) and Fluorescein (VEGFR-2) substrates were used in our analysis.

### Immunohistochemistry

Single systems of control and ampullaectomized *B. schlosseri* colonies were anesthetized using MS-222 (MP Biomedicals, 103106) until oral siphons opened and were unresponsive. Systems were then fixed with 4% paraformaldyhyde (Electron Microscopy Sciences, 15710) with 0.5 M NaCl at 4°C for 12 hours. Following fixation, systems were bleached using 6% H_2_0_2_ in methanol under light for 1 hour at room temperature to quench auto-fluorescence. Systems were then washed through a series graded methanol and PBS with 0.1% Tween-20 (PBT) and then blocked with 5% heat inactivated horse serum (Jackson ImmunoResearch, 008-000-001) with 2 mg/ml IgG-free BSA (Jackson ImmunoResearch, 001-000-162) in PBT at room temperature for 4 hours. Systems were then incubated with either a Rabbit pan-Cadherin antibody (Cell Signaling, 4068P) (1∶500) or Rabbit pHH3 antibody (Millipore, 06-570) (1∶1000) in blocking buffer and incubated for 48 hours at 4°C. Following primary incubation, system were washed with PBT and incubated with an Alexa Fluor 488 Goat Anti-Rabbit secondary antibody (Life Technologies, A-11008) (1∶200) for 24 hours at 4°C. Systems were then washed with PBT for 6 hours at room temperature to remove unbound secondary antibody and flat mounted in Vectashield mounting medium for fluorescence with DAPI (Vector Labs, H-1200).

### Imaging

Live imaging of single and chimaeric colonies was carried out using a MZ16FA (Leica) equipped with epifluorescence capabilities to image vascular labeling. Fixed tissue for both immunohisotchemistry and *in situ* hybridization was imaged on a 1000 Fluoview Spectral Confocal (Olympus).

### FACS and RNA extraction

*Botryllus schlosseri* vascular cells were isolated for sorting as outlined in previous studies (Laird, 2005). Briefly, animals were injected with 1 µl of 1 µg/ml FITC-Dextran per system. Cells were then extracted by mincing whole animals followed by 70 µm and 40 µm filtration into ice-cold *Botryllus* buffer (50 mM EDTA pH 8.0, 25 mM HEPES pH 7.5, 10 mM cysteine) in sterile seawater containing 2% isotonic horse serum (Jackson ImmunoResearch, 008-000-001). Cells were peleted at 500 g for10 minutes at 4°C and then resuspended in 500 µl of *Botryllus* buffer.

Fluorescently Activated Cell Sorting (FACS) was carried out using a FACSAria (BD Biosciences). Samples were manually compensated and gated as positive or negative based on uninjected control animal fluorescence. Analysis was the performed using BD FACS Diva software (BD Biosciences) and positive cells were sorted. Sorted cells were sorted directly into Lysis buffer for immediate RNA extraction. RNA was extracted using magnetic mRNA isolation kit (New England BioLabs, S1550S) and then reverse transcribed to cDNA using oligo (dT) primer (Life Technologies, AM5730G) and Superscript II Reverse Transcriptase (Life Technologies, 18064-014) according to manufacture guidelines.

### Quantitative RT-PCR

Quantitative RT-PCR (QRT-PCR) was carried out using a LightCycler 480 II (Roche) and LightCycler DNA Master SYBR Green I detection (Roche, 12015099001). The thermocycling profile was as follows: 5 minutes at 95°C, 45 cycles of 95°C for 10 sec, 50-60°C for 10 sec, and 72°C for 10 sec. Primers for CD133: 5′-atagtagatcacacatgtaaccccatc-3′ and 5′-tgtataatcagtaccgtgtgacaaaat-3′which amplified a 227 bp fragment, VEGFR-2 primers: 5′-gaagctttgatggatcgtaagatagcacc-3′ and 5′-agtcataatgcaactcgtttatctcaaagt-3′ which amplified a 234 bp fragment and Cadherin primers: 5′-ttttcctatcactgttttcaatttacc-3′ and 5′- aattttgacttcgatcatatactttgg-3′ which amplified a 179 bp fragment were used in our analysis. All gene expression data was normalized to elongation factor 1-α (EF1-α) as a reference gene and reported as relative expression using the 2^−ΔΔCt^ method. Each experiment was analyzed in triplicate from different genotypes (n = 3) and multiple systems (n = 4) for both control and ampullaectomy (AMPX) colonies.

## Results

### Vascular Tissue Exhibits Stochastic Regenerative Proliferation

**Figure 2:**
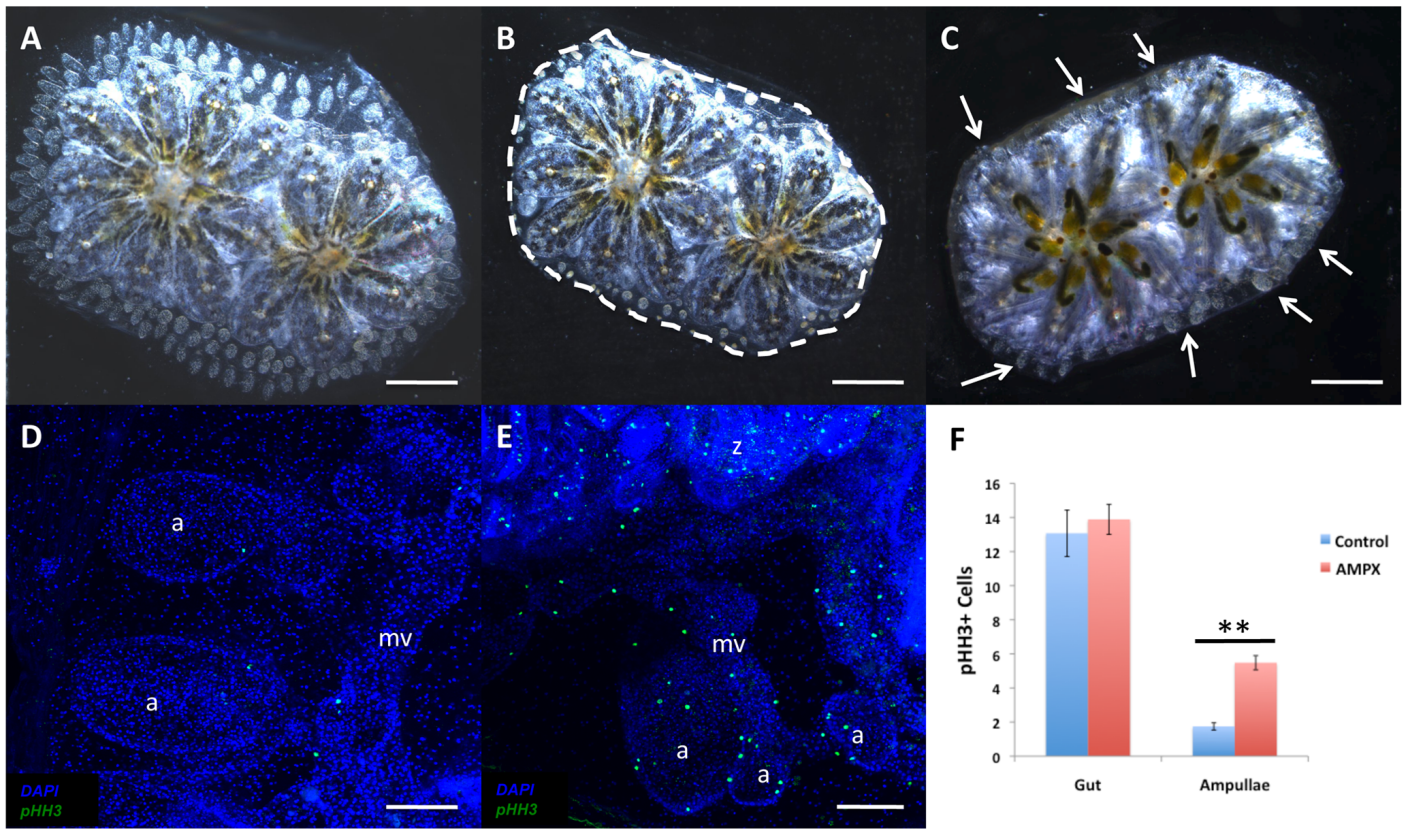
Cells within vascular tissue exhibit stochastic regenerative proliferation. (**A**) Dorsal view of adult *Botryllus schlosseri* colony prior to ampullaectomy surgery. (**B**) Dorsal view of the same colony immediately following ampullaectomy surgery (dashes) to remove peripheral ampullae and portions of the marginal vessel. (**C**) Ventral view of the same colony 24 hours post-ampullaectomy surgery with areas of regenerated ampullae (arrows). (**D–E**) Phospho-Histone H3 (pHH3) immunohistochemistry staining of peripheral ampullae (a) and marginal vessel (mv) in a control colony (**D**) and AMPX colony showing proliferation in the ampullae (a), marginal vessel (mv), and tissues of the zooid (z) (**E**). (**F**) pHH3 positive cell counts in gut and peripheral ampullae tissue in control and AMPX colonies (n = 6, * = p<0.05). Scale bars: A–C = 1 mm, D–E = 100 µm.

To characterize the cellular and molecular mechanisms regulating vascular regeneration in *Botryllus* we first sought to identify the source of new vascular tissue by analyzing the source of cell proliferation following surgical ablation of the extracorporeal vasculature compared to unmanipulated controls. Regeneration of vascular tissue is induced by removing the entire peripheral vasculature, consisting of marginal vessels and ampullae in a procedure known as an ampullaectomy (AMPX), as previously described [15]. We performed AMPX on adult developmentally stage-matched colonies (5–12 zooids, 3–6 months old, n = 6) (Figure 2A) and monitored them (Figure 2B) using time-lapse microscopy to observe vascular regeneration. The majority of vascular regeneration including marginal vessel remodeling and ampullae budding from the marginal vessels took place within the first 24 hours post-AMPX (Figure 2C) consistent with previous results [15].

The location and levels of cell proliferation were characterized using immunohistochemistry to identify the cell division metaphase marker phospho-Histone H3 (pHH3) in normal versus regenerating vascular tissue. In unmanipulated control vasculature, peripheral ampullae and marginal vessels were largely quiescent with a limited number of randomly distributed proliferating cells within blood vessels and peripheral ampullae in control animals (Figure 2D). However, during regeneration the number of actively dividing cells in these tissues significantly increased. The increase in actively dividing cells occurred in a stochastic unpredictable manner (Figure 2E). Peripheral ampullae showed a statistically significant (p<0.01 using Students t-test) increase in cellular proliferation at 24 hours post AMPX with an average of 5.48 pHH3^+^ cells per ampullae with an s.e.m. of 0.42 compared to control ampullae with an average of 1.75 pHH3^+^ cells per ampullae with an s.e.m. of 0.21 (Figure 2F).

To determine if the increase in vascular cell proliferation during regeneration was due to a global somatic proliferation response to injury or was a specific regenerative response by vascular tissue, we examined the number of proliferative cells in the gut of individual zooids in these colonies. We found that although the gut is a highly proliferative tissue there was no significant increase in cell proliferation between control (average of 13.07 pHH3^+^ cells in individual zooid gut with an s.e.m of 1.36) and AMPX (average of 13.89 pHH3^+^ cells in individual zooid gut with an s.e.m. of 0.88) colonies. The lack of proliferative response by another somatic tissue such as the gut coupled with the stochastic active cell proliferation of vascular tissue during vascular regeneration indicated that resident vascular cells were capable of regenerative proliferation following injury.

### Injection of pH-Stable Macromolecule Fluorophores Allows Vascular Cell Lineage Tracing

Following our observation that resident vascular cells exhibit stochastic regenerative proliferation, we next sought to determine the lineage of resident vascular cells contributing to the regeneration of both marginal vessels and ampullae in *Botryllus*. We developed a novel vascular cell lineage tracing technique that utilized both the endocytic properties of the *Botryllus* vasculature as well as the pH-stability of mCherry protein, FITC labeled dextran, and Alexa Fluor conjugated BSA to label vascular tissue.

**Figure 3:**
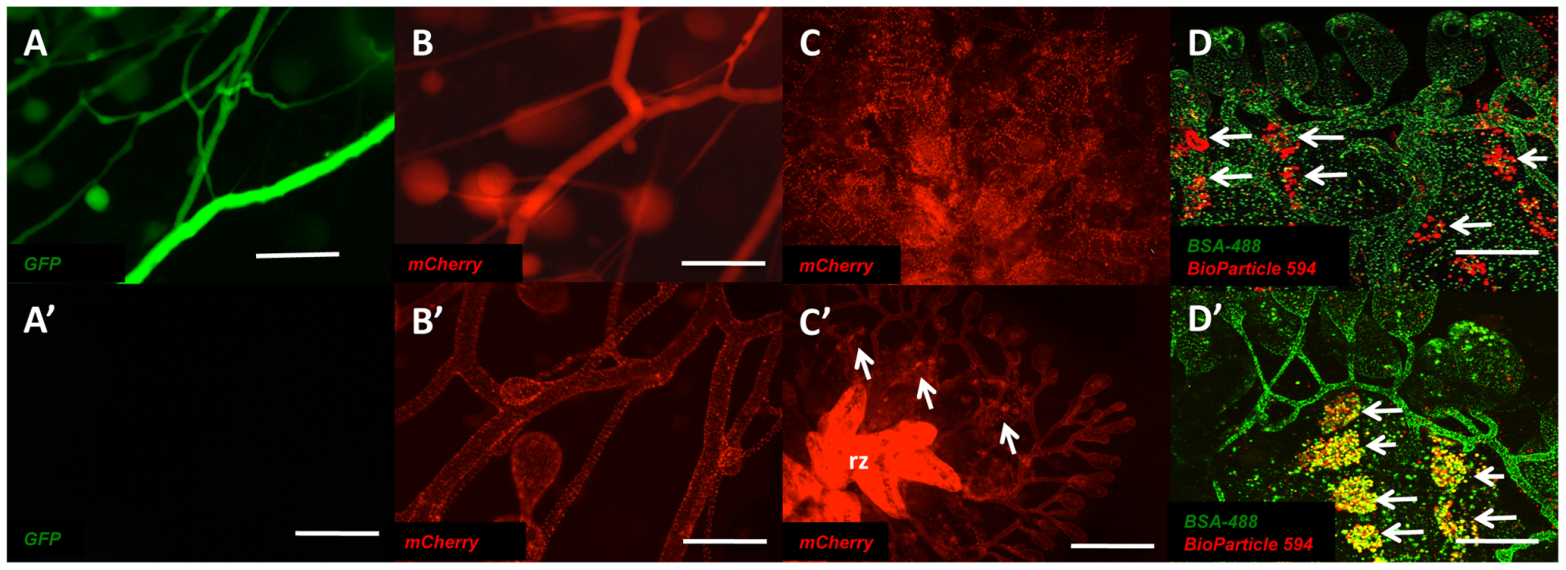
Vascular cell labeling is achieved though injection of mCherry protein but not GFP. (**A**) Ventral view of extracorporeal vasculature of *Botryllus schlosseri* immediately following injection of 1 µl of 0.1 µg/ml recombinant GFP protein into the circulation and (**A′**) 24 hours post injection. (**B**) Ventral view of extracorporeal vasculature of *Botryllus schlosseri* immediately following injection of 1 µl of 0.1 µg/ml recombinant mCherry protein into the circulation and (**B′**) 24 hours post injection. (**C**) Ventral view of mCherry labeled vasculature within a single system of *Botryllus schlosseri* and (**C′**) vascular labeling during a takeover event with regressing zooids (rz) and the appearance of mCherry labeling in ventral island phagocytes (arrows). (**D**) Ventral view of *Botryllus schlosseri* colony co-injected with Alexa Fluor 488 conjugated BSA and Alexa Fluor 594 conjugated Bio-Particles showing labeled vessels and phagocytic island populations (arrows). (**D′**) Same co-injected *Botryllus schlosseri* colony following apoptotic clearance of regressing zooids showing double labeling of phagocytic island populations (arrows). Scale bars: A–B = 200 µm, C = 800 µm, C′ = 1 mm, D = 800 µm.

The peripheral injection of fluorophores into the ampullae of *Botryllus* colonies has previously been used to fluorescently visualize the vasculature system of the colony [15]. While such injections allow for the immediate visualization of blood flow and the vascular network in *Botryllus* colonies, pH-unstable fluorophores such as bacterially expressed GFP can only be visualized within the first 24 hours post injection in the blood stream of these animals (Figure 3A). Within the first 24 hours post-injection, GFP is cleared from the blood of the colony and can no longer be used to visualize the extracorporeal vasculature (Figure 3A′). However, during that period of clearance, the epithelium of the vasculature became briefly labeled. We hypothesized that the vasculature tissue itself may be involved in the clearance of fluorophores such as GFP from the blood of the colony through both the endocytic and lysosomal activity of resident vascular cells. To test this hypothesis we injected a bacterially expressed mCherry protein that has the ability to maintain its fluorescent properties in low pH environments such as lysosomes [21], [22]. Similar to the injections of GFP, mCherry signal was visualized in the blood of the colony following injection into the peripheral ampullae (Figure 3B). However, unlike GFP, fluorescent signal from the mCherry protein could be visualized 24 hours post-injection in a punctate pattern within the vascular tissue (Figure 3B′). This punctate pattern of mCherry fluorescence was maintained in vascular tissue over an average of a 15-day period (+/− 2 days, n = 12) and was inert in terms of physiological response to the injection. In addition to mCherry protein we found that the injection of Alexa Fluors conjugated to BSA were also capable of vascular cell labeling at the same concentrations and provided increased fluorescence due to the brightness of the fluorophore.

In addition to this vascular tissue labeling, we also observed a number of phagocytic cells being labeled within previously described regions called ventral islands. This only occurred following a weekly event called takeover, which involves clearance regressing apoptotic tissue [23], [24]. Figure 3C shows that labeling of these phagocytic island populations is not seen prior to the takeover event, but can be observed following the phagocytic clearance of vascular cells in the regressing zooids (Figure 3C′) and these phagocytes maintained fluorescence for the duration of the mCherry labeling. To confirm that the fluorescence seen in these islands was co-localized with phagocytic cell populations we co-labeled colonies with fluorescently labeled BioParticles as previously described [24] and Alexa Fluor 488 conjugated BSA to label vessels (Figure 3D). In summary, the vasculature selectively takes up the injected protein, and when portions undergo apoptosis during takeover, the label is stably transferred to a population of phagocytic cells (Figure 3D').

**Figure 4:**
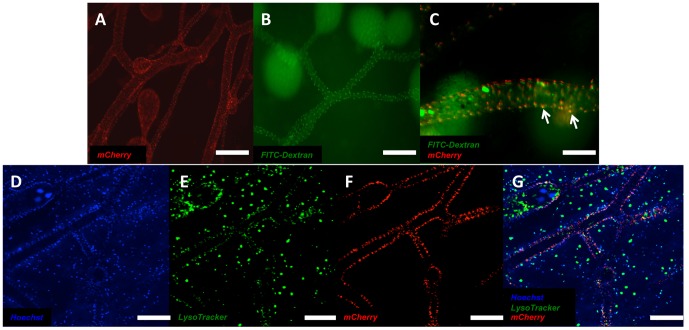
pH-stable macromolecule fluorophores are maintained in vascular lysosomes. pH-stable macromolecules fluorophores such as mCherry (**A**) and FITC-conjugated dextran (**B**) are able to be taken up by vascular tissue and label the same internal cellular structures (arrows) as seen in animals that are co-injected with FITC-conjugated dextran and mCherry (**C**). mCherry fluorescence is co-localized with lysosomes in *Botryllus schlosseri* vasculature cells as indicated by (**D**) Hoechst 33342 staining of marginal vessels and peripheral ampullae, (**E**) LysoTracker Green stain of these vessels, (**F**) uptake of mCherry in these vasculature tissues, and (**G**) the co-localization of mCherry and LysoTracker fluorescence. Scale bars: A–B = 200 µm, C = 75 µm, D–G = 200 µm.

To determine if the uptake of mCherry by the vasculature tissue of *Botryllus* was protein-specific we injected a FITC-conjugated dextran (FITC-dextran), a macromolecule that is also fluorescently stable in low pH environments, into the peripheral vasculature of *Botryllus* in a similar manner as the mCherry protein. We found that the injection of both mCherry protein (Figure 4A) and FITC-dextran (Figure 4B) had the ability to label vascular cells and when co-injected these fluorescent macromolecules labeled the same cellular structures (Figure 4C), and were similarly transferred to phagocytic cells following takeover.

Finally, we confirmed the intracellular location of the mCherry protein within vascular cells, and used LysoTracker Green to label lysosomes in mCherry-injected animals. Confocal analysis (Figure 4D–G) indicated the co-localization of mCherry signal with LysoTracker in vascular tissues. This specific labeling of vascular cell lysosomes using mCherry, FITC-Dextran or Alexa Fluor conjugated BSA gave us the ability to track lysosomes in actively dividing cells of the *Botryllus* vasculature, in turn allowing us to use this technique to trace vascular cell lineages during vascular regeneration.

### Resident Vascular Cells Exhibit Bi-Potentiality in Regenerating Vascular Tissue

**Figure 5:**
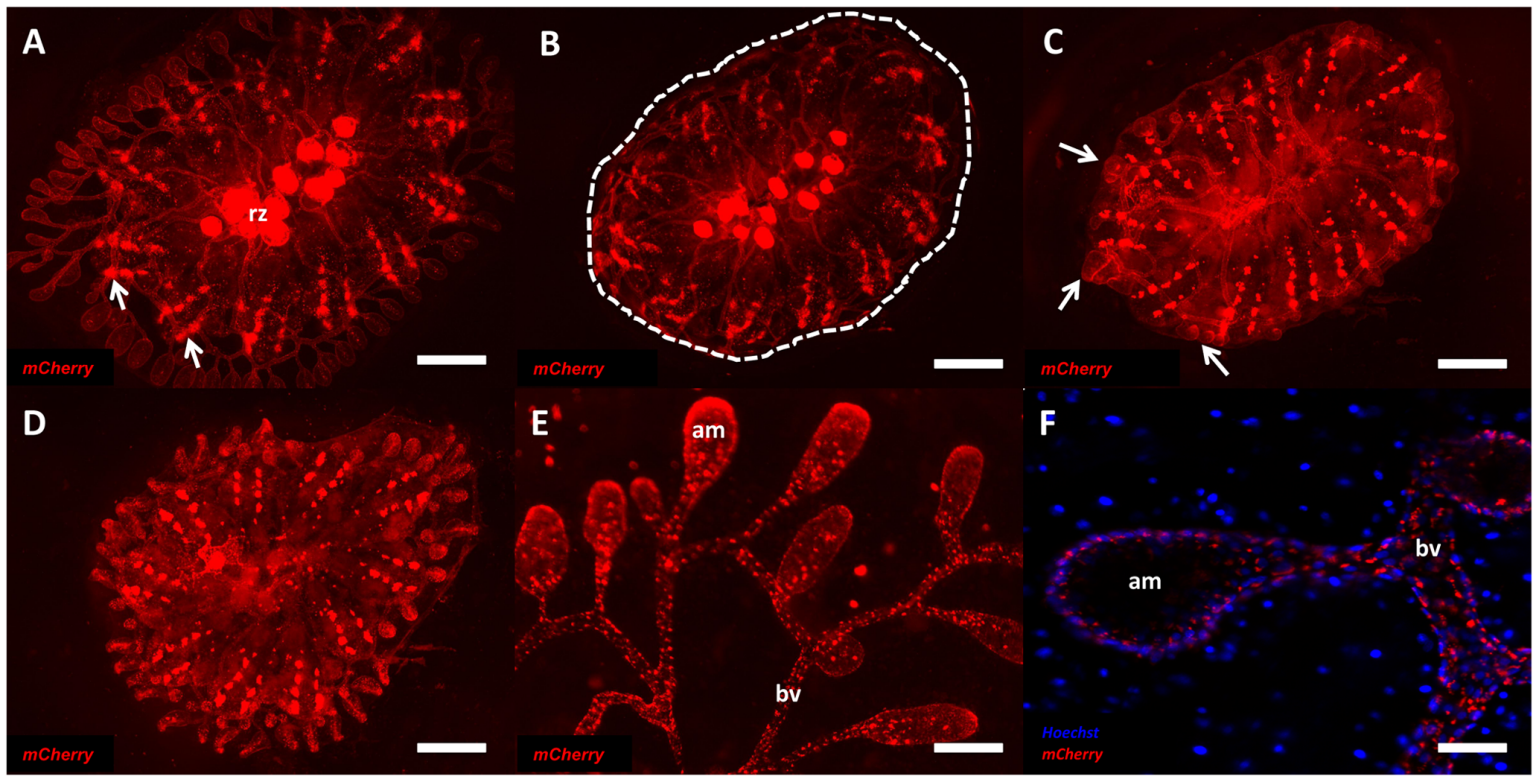
Resident vascular cells exhibit bi-potentiality during vascular regeneration. (**A**) Ventral view of a mCherry labeled *Botryllus schlosseri* system with labeled vasculature tissue, regressing zooids (rz) and labeled ventral phagocytic islands (arrows). (**B**) Ventral view of mCherry labeled system immediately flowing AMPX surgery (dots). (**C**) Ventral view of system 24 hours post AMPX showing regenerated labeled ampullae (arrows). (**D**) Ventral view of system showing complete regeneration of extracorporeal vasculature at 72 hours post AMPX. (**E**) Regenerated blood vessels (bv) and peripheral ampullae (am) showing mCherry labeling 72 hours post AMPX. (**F**) Live cell image of mCherry labeling inside cells of regenerated ampullae (am) and blood vessels (bv) counterstained with Hoechst 33342. Scale bars: A–D = 1 mm, E = 200 µm, F = 50 µm.

Utilizing our vascular cell lineage tracing technique we sought to answer the question of whether pre-existing vascular resident cells had the potential to contribute to cells of regenerating ampullae following AMPX. To answer this question we injected mCherry protein into the vasculature of *Botryllus* colonies (5–12 zooids, 3–6 months old, n = 6) (Figure 5A) to label the existing vascular tissue. Following a 24-hour period to allow for mCherry protein to be internalized, we performed an AMPX surgery to remove the peripheral vasculature and ampullae as well as portions of the medial vessel (Figure 5B). Within 24 hours post-AMPX, mCherry labeled regenerated vessels and ampullae were observed emerging from the pre-existing vasculature tissue (Figure 5C). The vascular network was completely regenerated within 48–72 hours following ampullaectomy, and mCherry labeling could be observed in all regenerated vascular tissues (Figure 5D). The mCherry labeled regenerated vascular tissue was observed in both peripheral vessels as well as regenerated ampullae at 72-hours post-AMPX (Figure 5E). To confirm that mCherry signal was present in all cells of the regenerating ampullae and that this signal was from cells within the vasculature epithelium we counterstained mCherry injected animals that had regenerated vasculature tissue with Hoechst 33342 to label nuclei. We observed that all cells within the regenerating vasculature epithelium contained mCherry signal suggesting that pre-existing vascular cells were the source of both the newly regenerated blood vessels and ampullae following surgical ablation of the tissue (Figure 5F).

### Resident Vascular Cells Differentiate through CD133 Expressing Intermediates

**Figure 6:**
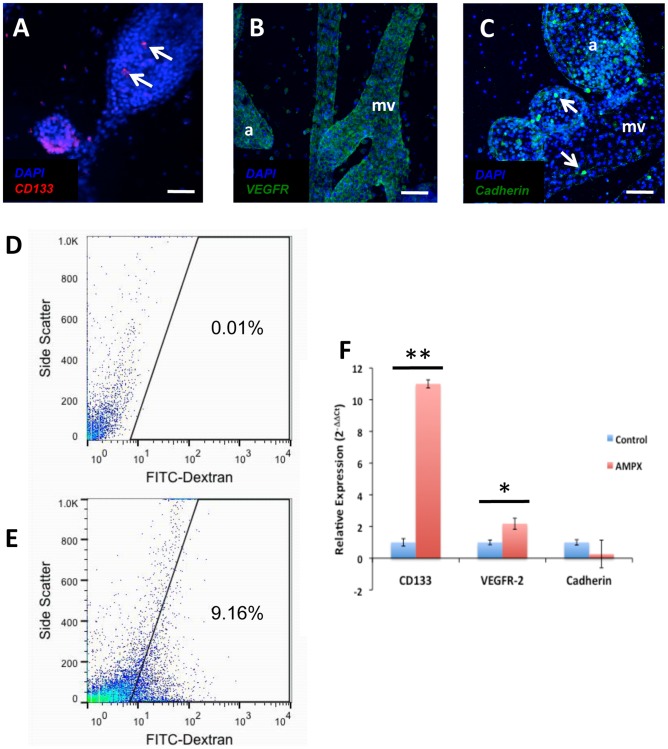
Resident vascular cells differentially express progenitor and differentiated markers during regenerating. (**A**) *In situ* hybridization of CD133 showing expression in developing ampullae and a small subset up blood cells (arrows). (**B**) In situ hybridization showing VEGFR-2 expression in both ampullae (a) and marginal vessels (mv). (**C**) Immunohistochemistry of Cadherin showing expression in developing ampullae (a), marginal vessels (mv), and blood cells (arrows). (**D**) FITC-Dextran fluorescence vs. Side Scatter FACS analysis showing the auto-fluorescence of unlabeled control animals of a *Botryllus schlosseri* whole cell isolation following mechanical cell dissociation (n = 12). (**E**) FITC-Dextran fluorescence vs. Side Scatter FACS analysis of FITC-Dextran vascular labeled animals (n = 12). (**F**) QRT-PCR analysis of FACS isolated FITC-dextran labeled vascular cells showing 11.0 (+/− s.e.m. = 0.26) fold up regulation of CD133, 2.2 (+/− s.e.m. = 0.35) up regulation of VEGFR-2 and 0.74 (+/− s.e.m. = 0.88) regulation of Cadherin in AMPX animals (n = 12) compared to stage matched controls (n = 12). * = p<0.01, ** = p<0.001. Scale bars: A = 50 µm, B = 70 µm, C = 50 µm.

Following our observation that labeled resident vascular cells had the potential to contribute to both regenerated blood vessels and ampullae, we sought to identify the molecular mechanisms by which these vascular resident cells differentiated into ampullae cells. Previous data suggested that *Botryllus* homologs of vertebrate genes, whose involvement in both vascular progenitors (CD133 and VEGFR-2) and differentiated vascular cell markers (VE-Cadherin) has been described in vertebrate vascular regeneration, may be regulated in *Botryllus* vascular resident cells during vascular regeneration. To test this hypothesis we used *in situ* hybridization for CD133 and VEGFR to localize these genes in regenerating vascular tissue. We found that CD133 was expressed by newly budding ampullae and a small subset of blood cells in *Botryllus* (Figure 6A). While expression of CD133 was observed in newly budding ampullae from the marginal vessel, the mRNA was not expressed in mature peripheral ampullae or the marginal vessel itself. Consistent with previous results [15] we also observed that VEGFR was expressed both by regenerating and mature ampullae as well as the marginal vessel (Figure 6B). In addition to the spatial localization of CD133 and VEGFR we used immunohistochemistry to localize Cadherin in the *Botryllus* vasculature tissue and found that consistent with previous results [25] Cadherin was expressed by regenerating and mature ampullae, the marginal vessel, and a variety of blood cells (Figure 6C).

Following the determination of the spatial expression of these genes we then analyzed the relative expression levels of *Botryllus* homologs of CD133, VEGFR, and Cadherin using qRT-PCR of vascular cells that had been isolated using fluorescently activated cell sorting (FACS) analysis. FACS was used to isolate labeled vascular cells in both control and regenerating conditions prior to takeover events to limit the amount of phagocytic cell labeling (Figure 6D–E). These isolated vascular cells were then analyzed for gene expression changes for *Botryllus* homologs of CD133, VEGFR, and Cadherin. qRT-PCR of these sorted vascular resident cells showed 11.0 (+/−0.26) fold up regulation of CD133 in AMPX animals, 2.2 (+/− 0.35) fold up regulation of VEGFR and 0.74 (+/− 0.88) fold down regulation of Cadherin in AMPX animals, (n = 3 genotypes, 4 systems each) when compared to the stage-matched controls (n = 3 genotypes, 4 systems each) (Figure 6F). Comparing ampullaectomy samples with controls, results showed significant up-regulation of CD133 and VEGFR-2 but not Cadherin by resident vascular cells during the regeneration of vascular tissue following surgical ablation of the tissue.

### Resident Vascular Cells Maintain Organismal Identity and Regeneration Potential in Parabiosed Chimeras

**Figure 7:**
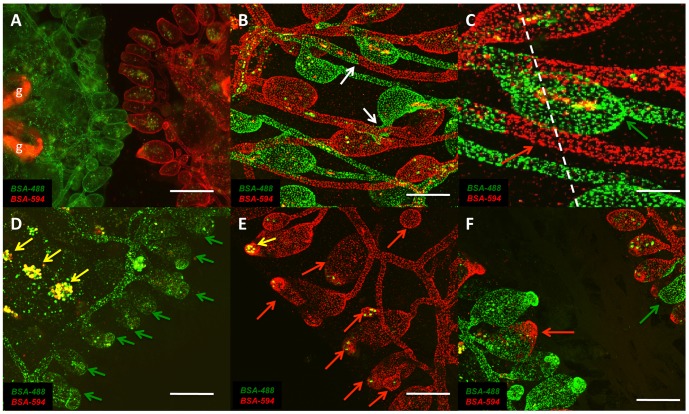
Resident vascular cells maintain individual identity in regenerating chimeras. (**A**) Ventral view of two *Botryllus schlosseri* colonies injected with Alexa Fluor 488 conjugated BSA (left) also showing auto-fluorescence in the gut (g) and a colony injected with Alexa Fluor 594 conjugated BSA (right) prior to vascular fusion of ampullae. (**B**) Ventral view of the chimeric colony vasculature following fusion (arrows) 48 hours after initial interaction of ampullae. (**C**) Ventral view of fused vasculature indicating surgical separation (dashes) of parabiosed vasculature to generate chimeric vessels with either Alexa Fluor 488 conjugated BSA (green arrow) or Alexa Fluor 594 conjugated BSA (red arrow) labeled distal tips. (**D**) Ventral view of colony injected with Alexa Fluor 488 conjugated BSA labeled vasculature showing blood chimerism in ventral islands (yellow arrows) and regenerating ampullae (green arrows) 24 hours post AMPX (**E**) Ventral view of colony injected with Alexa Fluor 594 conjugated BSA labeled vasculature showing blood chimerism in ampullae (yellow arrows) and regenerating ampullae (red arrows) 24 hours post AMPX. (**F**) Ventral view of surgically separated chimeric colonies 24 hours post AMPX showing chimeric ampullae regeneration and remodeling of chimeric vessels with Alexa Fluor 488 conjugated BSA (green arrow) and Alexa Fluor 594 conjugated BSA (red arrow) distal tips. Scale bars: A = 500 µm, B = 200 µm, C = 50 µm, D–F = 250 µm.

The contribution of pre-existing vascular resident cells and the differential regulation of vascular progenitor and differentiated vascular cell markers suggested the mobility of vascular cells contributing to the regeneration of vascular tissue in *Botryllus* might be limited. To assess the potential mobility of vascular cells during vascular regeneration we analyzed the cell lineage of regenerated vascular cells in parabiosed blood chimeras. To establish the chimeric vascular-labeled colonies needed for these experiments we injected sub-clones of genetically identical individuals with either Alexa Fluor 488 conjugated BSA or Alexa Fluor 594 conjugated BSA, waited 24 hours for vascular labeling to occur and then set up fusion assays as previously described [8], [10]. The parabiosis of the labeled colonies is shown in Figure 7, panels A-C. Figure 7A shows the initial contact of the two ampullae, and Figures 7B and C shows the vessels following fusion. During the parabiosis, multiple ampullae interact, with several fusing through vascular anastamosis. Following fusion of the ampullae, they remodel into a vessel, which involves down-regulation of the allorecognition proteins fuhc^sec^, fuhc^tm^, fester and uncle fester [8]–[10] expressed exclusively at the tips of the ampullae, but not on the vessels. A close-up of two fused vessels ca. 48 h following fusion is shown in Figure 7B, and reveals a very clear demarcation between the two labeled vasculatures (arrows).

Following the vascular anastamosis of the two separately labeled colonies, the parabiosed colonies were left for 3–5 days, allowing both colonies to undergo a takeover event. This allowed us to also label the phagocytic cells, which could then be used to confirm blood chimerism. At that point we surgically removed the vasculature at the base of each individual, as close to the zooids as possible (shown in Figure 5B), and monitored the regenerating vasculature. As shown in Figures 7D and 7E, the regenerated vasculature of each colony maintained its original labeling, as indicated by the distinct separation of BSA-Alexa Fluor 488 and BSA-Alexa Fluor 594 labeled vasculature cells. This demonstrates that individual specificity of the vasculature was maintained throughout the time-course of the experiment (7–10 days, n = 3 genotypes, 4 chimeric assays per genotype).

In contrast, the labeled phagocytic cells had transferred between the two individuals, and contained label of the other partner. In addition, we saw a number of double-labeled phagocytic cells, indicating they had engulfed dying cells in both individuals, consistent with previous result using double-labeled phagocytic populations [24].

In addition to the animal specific regenerative potential of blood vessels and ampullae, the lack of double-labeled vascular cells in these chimeric animals also confirmed that injected fluorophores used to label vascular tissue was not being recycled through phagocytic cells but rather the observed regenerative labeling arose from the contribution of specifically labeled resident vascular cells.

To further delineate the source of the differentiated ampullae, we repeated this experiment, but instead of cutting the ampullae at the base of each individual, we surgically separated the parabiosed individuals by slicing out a section across the median of the fused vessels, such that a subset of vessels was chimeric, with the distal portion being from one juxtaposed partner, and the proximal from the other (Figure 7C). We found that during remodeling, chimeric vessels formed chimeric ampullae, where the distal portion of the cut vessel had differentiated into the tip of the ampullae, while the proximal portion retained vessel or proximal ampullae identity (Figure 7F). In these experiments each cell retained its original label, as no individual double-labeled cells were ever observed, confirming that there is no evidence of label transfer during regeneration or remodeling of vascular tissue. In addition, there were no instances where the labeled portion had reversed orientation: all chimeric ampullae were labeled consistent with their location following the surgery.

In summary, these experiments reveal that individual cells adopt either vessel or ampullae fates depending on their original location following surgical manipulation, with the distal portion becoming the distal tip of the ampullae, and the proximal region retaining vessel or proximal ampullae identity. Thus, immobile, resident vascular cells regenerate new vessels and have the ability to reversibly switch between vessel and ampullae cell fates during vascular remodeling events.

## Discussion

Identifying the source of regeneration of the *Botryllus* vasculature is fundamental to understanding how these basal chordates regenerate and maintain differentiated vascular tissue involved in colony growth, adherence and allorecognition capabilities. Through a novel vascular cell lineage tracing method we show that the regenerative proliferation of resident cells within the vascular tissue of *Botryllus* have the potential to contribute to both regenerated blood vessels as well as regenerated differentiated vascular tissue known as ampullae. These cells express both progenitor and differentiated cell markers including the *Botryllus* homologs of CD133, VEGFR-2, and Cadherin during the vascular regenerative process and represent an immobile cell population in chimeric animals that maintain individual tissue identity during vascular regeneration. Furthermore, these cells can reversibly adopt two differentiated phenotypes, those of the vessel wall, and those of the ampullae, which can be differentiated by the expression of four allorecognition proteins.

Following injury, early signaling events stimulate the production of additional cells that are capable of rebuilding lost tissues [1], [26], [27]. The production of additional cells can be generated in a variety of ways, including proliferation of a resident stem cell population, division of terminally differentiated cells, or dedifferentiation/transdifferentiation of mature cells to a stem cell-like fate [1]. In *Botryllus*, we found that the production of additional cells contributing to the regeneration of vascular tissues occurs through the stochastic proliferation of pre-existing vascular resident cells. This stochastic regenerative proliferation indicates that the regenerative potential of this tissue is due to the proliferation and differentiation of pre-existing vascular resident cells, rather than a regenerative proliferation within a specific niche of undifferentiated vascular resident stem cells. Such an increase in the stochastic proliferation observed in pre-existing vascular resident cells suggests that the regenerative potential of vascular tissue in *Botryllus* occurs through an epimorphoric regenerative process where cells within the vascular tissue maintain the potential to both proliferate and differentiate. However, while there is no canonical spatial pattern to proliferation, this does not address whether there is still heterogeneity within the vascular cell population. The same can be said for differentiation between vascular and ampullae tissue, while the creation of chimeric ampullae indicates that pre-existing vascular cells have the potential to differentiate into ampullae, there still could be only a minor population of labeled vascular resident cells that do so.

While the stochastic proliferation of pre-existing vascular resident cells suggested that these cells were capable of rebuilding surgically ablated blood vessels and ampullae, confirming resident vascular cells as the source of vascular regeneration required cell lineage tracing. Here we show that the use of pH-stable macromolecules can be used as an *in vivo* method for cell lineage tracing in vascular tissue through lysosomes. Fluorophores that exhibit pH-stability have been used extensively to study lysosomal protein activity because of their fluorescent stability in low pH environments [21], [22]. It has been previously shown that as cells undergo mitosis, lysosomal partitioning occurs through an ordered process that results in the lysosomal copy number being maintained by daughter cells [28]. Such a mitotic distribution of lysosomes in conjunction with the ability to label these organelles in the *Botryllus* vasculature allows for the tracking of pre-existing vascular resident cells and their mitotic contributions to regenerating tissue. While it has yet to be determined what the specific uptake mechanism of these macromolecules is for vascular cells, it is clear that vascular cells in *Botryllus* have the ability to preferentially uptake macromolecules that have been introduced into the blood stream and provide a novel way to trace vascular cell lineages in this animal.

Using this vascular cell lineage tracing technique we were able to identify pre-existing vascular tissue resident cells as the source of vascular regeneration in *Botryllus* and observed the contribution of these cells to both regenerating blood vessels and differentiated ampullae tissue. Similar to *Botryllus*, the identification of pre-existing tissue resident cells as the source of regeneration and their lineage restriction following injury has been observed in a number of regenerating tissues in vertebrates [29]–[32]. For example, lineage-tracing experiments indicate that after damage to the zebrafish heart, existing cardiomyocytes undergo dedifferentiation and proliferation to generate new cardiomyocytes for replacing lost heart tissue [33], [34]. Liver progenitor cells also appear to be the major sources of new hepatocytes under conditions of extreme damage or chronic disease [35]. While such tissue resident cells are the source of proliferative regeneration in these tissues, many identified sources of regeneration are often lineage restricted [2], [29], [32], [36]. Such lineage restriction of sources of regeneration can be observed during limb regeneration in the axolotl. During limb regeneration each tissue produces progenitor cells with restricted potential indicating that limb regeneration can be achieved without complete dedifferentiation to a pluripotent state [30]. Additionally tissue resident cells do not switch between embryonic germ layers and most cell types are largely restricted to their own tissue identity during regeneration of the mouse digit tip [37]. Similarly, the vascular resident cells of *Botryllus* also seem to be lineage-restricted, as we observed that vascular cells solely gave rise to ectodermally derived blood vessels and ampullae. However the ability of these pre-existing blood vessel cells to differentiate into ampullae cells that express allorecognition proteins indicates that these vascular resident cells maintain lineage-restricted bi-potentiality in *Botryllus*.

While the source of regeneration and lineage potential has been established for a number of regenerating vertebrate tissues such as the skin, liver, and kidney, the source of neo-vascularization in vertebrates is still largely debated [37]–[40]. In the context of regeneration, the identity of neo-vascularization is of paramount importance due to the inability of regenerating tissue to sufficiently regenerate without adequate vascularization [41]–[43] While a conclusive source of regeneration for vascular tissue is unknown in the vertebrates, there is evidence for both mobile and resident cellular contributions to newly forming vasculature [38], [39], [44]–[46].

In mammals, the mobile vascular progenitor hypothesis first arose from studies showing that cells isolated from peripheral blood using CD34 and VEGFR-2 antibodies had the potential to differentiate into mature endothelial cells *in vitro* [47]. This data suggested that these isolated cells could contribute to neo-vascularization in adult species, previously only observed during developmental vasculogenesis. Subsequent studies on endothelial progenitor cells argued that bone marrow derived peripheral blood cells expressing a CD34^+^CD133^+^VEGFR-2^+^ molecular signature were able to home to and differentiate at sites of neo-vascularization suggesting the presence of mobile endothelial progenitor cells [48]. Additional studies on these cells suggested that as endothelial progenitor cells mature they lose expression of CD133 and begin to express the vascular endothelial marker VE-Cadherin expressed by mature differentiated vascular tissue [49]. The clinical relevance of these endothelial precursor cells has been shown through the demonstration that such bone marrow derived endothelial progenitor cells can restore tissue vascularization after ischemic events in limbs, retina and myocardium [42]. Such studies were further supported by evidence that the level of endothelial progenitor cells in blood circulation from high-risk cardiovascular disease subjects were both fewer in number and become senescent more rapidly than those from low-risk subjects [44].

While the above studies suggested that mobile vascular progenitors have been implicated in neo-vascularization, other studies have shown that no bone marrow derived VEFGR-2^+^ precursors contribute to the vascular endothelium and that neo-vascularization does not require bone marrow-derived endothelial progenitors [50]. For example, in the regeneration of the mouse distal digit a wide range of tissue stem/progenitor cells contribute toward the regeneration of this structure. However, the transplantation of hematopoietic stem cells, and parabiosis between genetically marked mice, confirmed that the stem/progenitor cells were tissue resident, including cells involved in the neo-vascularization of the regenerated structure. From these results it was argued that the angiogenesis responsible for vascularizing digit tips must be derived from an immobile population, not circulating hematopoietic or endothelial progenitors. Such studies suggest that resident tissue stem/progenitor cells are an evolutionary conserved cellular mode of regeneration of the vasculature [30], [37], [51].

Within the context that tissue resident cells may be evolutionary conserved units of regeneration we sought to determine if the *Botryllus* homologs for genes implicated in vertebrate endothelial neo-vascularization were also regulated during the regeneration of ectodermally derived vascular tissue in this basal chordate. While we were unable to identify a homolog to CD34 in *Botryllus*, homologs for CD133, VEGFR-2, and VE-Cadherin were expressed by the *Botryllus* vasculature. In congruence with previous studies on the regeneration of *Botryllus* vascular tissue [15] we observed an up-regulation of VEGFR-2 in isolated resident vascular cells during regeneration, confirming that migration of pre-existing vascular cells within existing vascular tissue play a fundamental role during the vascular regenerative process. Previous data describing *Botryllus* vascular regeneration showed that ampullae injury caused local increase in Cadherin protein in damaged epithelial tissue, a reaction that resembled expression patterns of endothelial VE-Cadherin during injury of normal blood vessel [25], [52]. While expressed by regenerating vascular tissue, our analysis showed a non-significant down regulation trend of Cadherin expression in isolated resident vascular cells 24 hours post-AMPX.

While both regenerating and mature blood vessel and ampullae tissue expressed VEGFR-2 and Cadherin, we found that CD133 was only expressed by newly forming/regenerating ampullae and a small subset of blood cells. In addition, CD133 was also significantly up-regulated in isolated resident vascular cells during regeneration, suggesting that CD133 may be expressed by newly forming ampullae during the regenerative process as they differentiate from blood vessels to ampullae. While CD133 was originally described as a somatic stem cell marker [53], the gene is also expressed in differentiated somatic tissues such as glandular epithelia [54]. These studies have brought into question whether CD133 is an appropriate marker to identify somatic stem cells or tissue progenitors. However, such data does not preclude the possibility that cells expressing CD133 may be differentiating in some manner. For example, the loss of CD133 has been associated with reduced ductal branching in the mammary gland suggesting its importance in regulating branching morphogenesis [55]. In addition to its proposed role in branching morphogenesis, CD133 has also been shown to play an active role in cell growth through its ability to interact and potentiate the anti-apoptotic and pro-angiogenic activities of VEGF in primary human endothelial cells [56]. The differential and spatial regulation of CD133 by pre-existing vascular cells in newly forming ampullae suggests a role for CD133 in *Botryllus* ampullae differentiation from blood vessels.

The regulation of VEGFR-2, Cadherin, and CD133 by resident vascular cells in an ectodermally derived vascular system suggests the potential for the conservation of the regulation of genes involved in endothelial angiogenesis in vertebrates during vascular regeneration in a basal chordate. It has been suggested that endothelial angiogenesis in vertebrates and the evolution of the vertebrate endothelial vascular system arose through the cooption of a hypoxia-sensing mechanism shown to be involved in branching morphogenesis in invertebrates [57], [58]. It has also been hypothesized that the FGF/FGFR axis seen in invertebrate branching morphogenesis of ectodermally derived tubes such as the developing *Drosphila* trachea system was replaced by a VEGF/VEGFR-mediated endothelial vascular system in the vertebrates [57]. Additionally it has been suggested that the endothelial cell-lined blood vessels of vertebrates may have been derived from invertebrate haemocytes or blood cells that formed intercellular contacts [59]. While the evolution of the vertebrate vascular system remains under investigation, the conservation of regenerative mechanisms, such as the regulation of VEGFR-2 and CD133 expression suggests roles for tissue migration and differentiation of pre-existing vascular resident cells durin*g* vascular regeneration in *Botryllus*.

While the study of vascular regeneration and the conservation of regenerative mechanisms in a basal chordate have implications into the evolution of the vertebrate vascular system, the identification of the source of vascular regeneration has additional significance in the context of the *Botryllus* allorecognition system. The highly polymorphic allorecognition system in *Botryllus* likely exists to strictly limit the possibility of vascular anastamosis, as stem cells in blood-based chimeras can move from one individual to the next, and once transplanted, these stem cells are in competition for contribution to somatic and germline tissues [7], [60], [61]. On both a cellular and anatomical level, parasitism is restricted to colonies capable of vascular anastamosis thus making the tips of ampullae, which express the proteins involved in the allorecognition response, act as the gatekeepers to the invasion of parasitic stem cells [7]. In the regenerating vasculature of *Botryllus* blood chimeras we observed that the regeneration of ampullae tissue maintained individual identity, thus while a fusion event can result in a chimeric germline for one of the individuals, it will not result in a chimeric vasculature. It may be that evolutionary pressures also played a role in limiting the source of vascular regeneration to an immobile precursor.
